# Association Between Masticatory Performance and Nutritional Risk in Older Adults Wearing Removable Dentures: A Cross-Sectional Study Using Composite Nutritional Indices

**DOI:** 10.3390/jcm15187074

**Published:** 2026-09-11

**Authors:** Toshiro Hirai, Yuka Abe, Ranko Kawata, Yuriko Kusumoto, Haruka Sako, Takashi Matsumoto, Deepesh Kumar Gupta, Kazuyoshi Baba

**Affiliations:** 1 Department of Prosthodontics, Graduate School of Dentistry, Showa Medical University, Tokyo 145-8515, Japan; 2 Division of Prosthodontics, Department of Prosthodontics, School of Dentistry, Showa Medical University, Tokyo 145-8515, Japan; 3 Department of Prosthodontics, Government Dental College, Raipur 492001, Chhattisgarh, India

**Keywords:** aged, malnutrition, masticatory performance, removable denture

## Abstract

**Background/Objectives**: Impaired masticatory function may compromise nutritional status in older adults wearing removable dentures, but its associations with nutritional risk indices remain unclear. This study aimed to examine associations between masticatory performance and risk defined by the Geriatric Nutritional Risk Index (GNRI), Prognostic Nutritional Index (PNI), and Controlling Nutritional Status (CONUT) score. **Methods**: This cross-sectional study included 179 outpatients aged ≥60 years wearing removable dentures. Masticatory performance was quantified by glucose concentration after chewing a gummy jelly. GNRI-defined risk was the prespecified primary outcome; PNI- and CONUT-defined risk were secondary. Logistic regression used Model 1 adjusted for age, sex, and comorbidity and an exploratory Model 2 with additional oral, dietary, and socioeconomic covariates. **Results**: Mean age was 74.1 ± 6.9 years, and 60.9% were female. Nutritional risk was identified in 21.8%, 48.6%, and 31.3% using the GNRI, PNI, and CONUT, respectively. Higher masticatory performance, indicated by higher glucose concentration, was associated with lower odds of GNRI-defined nutritional risk in both the primary parsimonious model (OR per 10 mg/dL increase, 0.89; 95% CI, 0.82–0.95; *p* = 0.001) and the exploratory additionally adjusted model (OR, 0.89; 95% CI, 0.82–0.96; *p* = 0.002). Associations with PNI- and CONUT-defined risk were not consistent across models. **Conclusions**: Lower masticatory performance was consistently associated with higher GNRI-defined risk, whereas comparable associations were not consistently observed for PNI- or CONUT-defined risk. Objective assessment of masticatory performance may provide clinically relevant information regarding GNRI-defined nutritional risk in older removable denture wearers.

## 1. Introduction

Tooth loss and impaired masticatory function are common in older adults and have been associated with adverse health outcomes and poorer quality of life [1]. Reduced chewing ability may limit the consumption of hard and fibrous foods [2,3], decrease dietary diversity, and contribute to imbalanced nutrient intake [4,5]. Impaired oral function has also been associated with social withdrawal [6], and tooth loss has been associated with loneliness in later life [7], as well as with an increased risk of physical frailty and sarcopenia [8].

Older adults who wear removable dentures are a clinically important population in this context because denture status is among the oral processing factors associated with nutrient intake [9]. A major goal of prosthodontic treatment is to restore masticatory function impaired by tooth loss [10]. Removable partial and complete dentures are frequently used in older adults because they provide a less invasive and generally less costly alternative to fixed prostheses [11]. Tooth loss has been associated with poorer nutritional status, primarily assessed using Mini Nutritional Assessment (MNA)-based tools [12], and, in a separate line of evidence, prosthodontic tooth replacement has been shown to influence nutritional status as assessed by the MNA [13]. However, evidence remains limited regarding the association between objectively measured masticatory performance and composite nutritional risk indices in older adults wearing removable dentures.

The Global Leadership Initiative on Malnutrition (GLIM) criteria provide a consensus framework for diagnosing malnutrition in adults by integrating phenotypic and etiologic criteria [14]. However, GLIM diagnosis requires information beyond routine blood biomarkers, including weight loss, reduced muscle mass, reduced food intake, and disease burden or inflammation. Composite indices based on routinely available clinical and laboratory data may therefore provide a practical means of identifying nutrition-related risk. Serum albumin has traditionally been used as a nutritional marker and has been associated with the number of remaining teeth and masticatory function [15,16]. However, because albumin is a negative acute-phase reactant, its serum concentration may decrease in response to inflammation independently of nutritional intake [17].

Several composite indices have been developed to address the limitations of using serum albumin alone. The Geriatric Nutritional Risk Index (GNRI) combines serum albumin with body-weight status [18], the Prognostic Nutritional Index (PNI) combines serum albumin with peripheral blood lymphocyte count [19,20], and the Controlling Nutritional Status (CONUT) score combines serum albumin, lymphocyte count, and total cholesterol [21]. Because these indices incorporate different components, they may reflect partly different dimensions of nutrition-related risk and may not classify the same individuals as being at risk. Previous studies in older adults with impaired oral function have generally examined only one nutritional index. To our knowledge, no study has simultaneously evaluated multiple composite nutritional risk indices in relation to objectively assessed masticatory performance in older adults wearing removable dentures.

Accordingly, this cross-sectional study aimed to examine the association between objectively assessed masticatory performance and GNRI-defined nutritional risk in older adults wearing removable dentures. GNRI was prespecified as the primary outcome because it was developed specifically for nutritional risk assessment in older adults. The secondary aims were to explore the associations of masticatory performance with PNI- and CONUT-defined nutritional risk and to describe the pairwise agreement among the three nutritional risk classifications.

## 2. Materials and Methods

### 2.1. Study Design and Setting

This single-center cross-sectional study was conducted at the Department of Prosthodontics, Showa Medical University Dental Hospital, Japan. Consecutive eligible outpatients were recruited between August 2023 and July 2024.

The study was conducted in accordance with the Declaration of Helsinki and was approved by the Institutional Review Board of Showa Medical University (protocol code 2023-108-A; approved on 1 August 2023). Informed consent was obtained from all participants. The manuscript was prepared in accordance with the Strengthening the Reporting of Observational Studies in Epidemiology (STROBE) Statement.

### 2.2. Study Population

Patients were eligible if they were aged ≥60 years and had worn removable dentures for at least one month [22,23]. Consecutive recruitment was used to reduce selection bias.

The exclusion criteria were receipt of dietary intervention within the previous six months, a pragmatic and conservative exclusion window selected to minimize potential residual effects of recent dietary counseling on nutritional status [24]; residence in or attendance at a nursing home; active malignancy; renal failure, nephrotic syndrome, hepatic failure, liver cirrhosis, or acute inflammatory disease; cognitive impairment that prevented adequate understanding or completion of the self-administered questionnaire; and use of an implant-supported overdenture.

Participants for whom the GNRI could not be validly calculated, or who had missing data for one or more variables required for the multivariable analyses, were excluded from the complete-case analytic sample. Participant flow, including the number screened, excluded, and analyzed, is reported in the Section 3.

### 2.3. Data Collection and Measurements

#### 2.3.1. Patient Characteristics

Demographic, clinical, dietary, and socioeconomic characteristics were collected at the study visit. These included age, sex, body mass index (BMI), Charlson Comorbidity Index (CCI) [25], living arrangement, perceived economic status, and estimated daily energy intake [26].

Age was recorded in years at the time of examination, and sex was recorded as male or female. Body weight was measured at the study visit, whereas height was self-reported. BMI was calculated as body weight in kilograms divided by height in meters squared. Comorbidity burden was assessed using the standard (not age-adjusted) CCI [25]. Because relatively few participants had comorbid conditions, CCI scores were categorized as 0 or ≥1.

Estimated daily energy intake was assessed using the brief-type self-administered diet history questionnaire (BDHQ) [26]. Participants reported the frequency of food and beverage consumption during the preceding month using a multiple-choice format, from which daily energy intake was estimated in kilocalories per day.

Living arrangement was categorized as living alone or living with others. Perceived economic status was assessed using an original self-administered question, a similarly worded item having also been used in a previous study [27]: “How do you currently feel about your degree of financial comfort?” Participants selected one of four response options: “very comfortable,” “somewhat comfortable,” “not very comfortable,” and “not comfortable at all.” Responses of “very comfortable” and “somewhat comfortable” were classified as high perceived economic status, and “not very comfortable” and “not comfortable at all” were classified as low perceived economic status.

#### 2.3.2. Oral Assessment

Masticatory performance was objectively assessed using the gummy jelly test [28]. Participants were instructed to chew a standardized gummy jelly (Glucolumn; GC Corp., Tokyo, Japan) for 20 s with their removable dentures in place. The gummy jelly and 10 mL of water were then expectorated through a dedicated filter into a collection cup. The glucose concentration in the filtrate was measured using a Gluco Sensor GS-II (GC Corp., Tokyo, Japan), based on a single measurement. Higher glucose concentrations indicated better masticatory performance. Consistent with previous studies assessing masticatory performance using a single measurement without a preceding habituation trial [29,30], the present study did not include a practice chewing trial before the test measurement.

The number of functional teeth was determined by intraoral examination. Functional teeth, defined as natural or restored teeth excluding those replaced by removable dentures, were counted based on a definition modified from a previous study [31], and included teeth with minor caries, fillings, or crowns, and abutment or pontic teeth of fixed prostheses, including bridges and implant-supported prostheses. Residual roots, teeth with Miller grade III mobility that were indicated for extraction, third molars without functional occlusal contact, and teeth replaced by removable dentures were not counted as functional teeth.

Denture quality was assessed by one of two trained prosthodontists (R.K. or H.S.) on the basis of denture stability and esthetics [32]. Each domain was rated using a 100 mm visual analog scale, anchored at 0 (“very poor”) and 100 (“very good”). The mean of the stability and esthetics scores was calculated as the overall denture quality score [33]. For participants wearing removable dentures in both the maxilla and mandible, each denture was assessed separately, and the lower of the two arch-specific overall scores was used in the analysis. Previous reliability testing by the same research group yielded an inter-rater intraclass correlation coefficient (ICC) of 0.85 (95% confidence interval [CI], 0.63–0.95) and a test–retest ICC of 0.92 (95% CI, 0.76–0.98) [34].

#### 2.3.3. Nutritional Assessment

Nutritional risk was assessed using the Geriatric Nutritional Risk Index (GNRI) [18], Prognostic Nutritional Index (PNI) [19,20], and Controlling Nutritional Status (CONUT) score [21]. Serum albumin concentration, peripheral blood lymphocyte count, and total cholesterol concentration were measured using fasting blood samples.

Participants were instructed to fast for at least 8 h before blood sampling, during which only water was permitted. Blood samples were collected in the morning and analyzed by a certified clinical laboratory (BML, Inc., Tokyo, Japan). Oral and dental assessments were performed within approximately 1–2 weeks of blood sampling. This interval was determined pragmatically based on the scheduling of blood sampling appointments, and no clinically meaningful change in oral status was expected within this short period.

The GNRI was the prespecified primary nutritional risk index. It was calculated according to the original formula [18]:

GNRI = (14.89 × serum albumin [g/dL]) + (41.7 × current body weight/ideal body weight)

Ideal body weight was calculated using the Lorentz formula:

Male: height (cm) − 100 − [(height [cm] − 150)/4]

Female: height (cm) − 100 − [(height [cm] − 150)/2.5]

When the ratio of current body weight to ideal body weight exceeded 1.0, the ratio was set to 1.0, in accordance with the original GNRI method. Participants were classified as having no nutritional risk when the GNRI was >98 and as being at nutritional risk when the GNRI was ≤98 [18].

The PNI was calculated using the equation proposed by Onodera et al. [19]:

PNI = (10 × serum albumin [g/dL]) + (0.005 × peripheral blood lymphocyte count [/mm^3^])

The PNI was originally developed to predict postoperative complications and prognosis in patients undergoing gastrointestinal surgery [19,20] but has subsequently been applied as a nutritional risk index in other populations, including older adults [35]. Based on previous studies [35,36], participants were classified as having no nutritional risk when the PNI was >50 and as being at nutritional risk when the PNI was ≤50.

The CONUT score was calculated from serum albumin concentration, peripheral blood lymphocyte count, and total cholesterol concentration according to the original scoring system [21]. Scores range from 0 to 12, with higher scores indicating greater index-defined nutritional risk. The standard categories are normal nutritional status, 0–1; mild undernutrition, 2–4; moderate undernutrition, 5–8; and severe undernutrition, 9–12 [21]. For the present analyses, participants were dichotomized according to the standard classification as having no nutritional risk (CONUT 0–1) or at nutritional risk (CONUT ≥ 2).

### 2.4. Sample Size

This study was designed as an exploratory cross-sectional study, and no formal a priori sample size calculation was performed. The sample size was determined pragmatically by the number of eligible patients who could be consecutively recruited during the prespecified study period. Because the number of GNRI-defined nutritional risk events was limited, the primary analysis used a parsimonious model, whereas the additionally adjusted model was considered exploratory, and Firth’s penalized logistic regression was performed for the primary model to assess the potential influence of sparse events (see Section 2.5).

### 2.5. Statistical Analysis

Statistical analyses were primarily performed using EZR version 1.70 (Jichi Medical University, Saitama, Japan), a graphical user interface for R. Additional analyses were conducted using R version 4.5.2 (R Foundation for Statistical Computing, Vienna, Austria).

Analyses were restricted to participants with complete data for all variables required for the multivariable analyses (complete-case analysis). The distributions of continuous variables were assessed using the Shapiro–Wilk test. Continuous variables are presented as mean ± standard deviation (SD) or median and interquartile range (IQR), as appropriate, and categorical variables as number and percentage.

For unadjusted comparisons between participants with and without GNRI-defined nutritional risk, continuous variables were compared using Student’s *t*-test or the Mann–Whitney *U* test, according to their distributions. Categorical variables were compared using the chi-square test or Fisher’s exact test, as appropriate.

Associations between masticatory performance and index-defined nutritional risk were examined using multivariable binary logistic regression. GNRI-defined nutritional risk was the prespecified primary outcome, whereas PNI- and CONUT-defined nutritional risk were secondary outcomes. Nutritional risk was coded as 1 and no nutritional risk as 0.

The primary explanatory variable was masticatory performance, assessed as the glucose concentration obtained from the gummy jelly test and analyzed as a continuous variable. Masticatory performance and estimated energy intake were rescaled by dividing their values by 10 and 100, respectively. Accordingly, odds ratios (ORs) represent a 10 mg/dL increase in masticatory performance or a 100 kcal/day increase in estimated energy intake. ORs, 95% CIs, and two-sided *p*-values were calculated.

Two hierarchically adjusted models were fitted for each outcome. Model 1, the primary parsimonious model, included masticatory performance, age, sex, and CCI. Model 2 additionally included denture quality, number of functional teeth, estimated energy intake, living arrangement, and perceived economic status. Because some of the additional variables in Model 2 may represent intermediate factors rather than exclusively confounding variables, Model 2 was regarded as exploratory and was not interpreted as estimating a confounder-independent association.

Multicollinearity was assessed using variance inflation factors (VIFs), with values <5 considered to indicate no substantial multicollinearity. The linearity of the association between each continuous explanatory variable and the logit of the outcome was assessed using the Box–Tidwell approach, in which an interaction term between each continuous variable and its natural logarithm was added to the corresponding logistic regression model [37]. Because the number of functional teeth included seven participants with a value of zero, for whom this transformation is undefined, functional teeth were instead evaluated using a sensitivity analysis in which this variable was categorized into four groups (0, 1–9, 10–19, and ≥20 functional teeth), modified from categories used in a previous study of Japanese older adults [31], and Model 2 for each outcome was refitted accordingly. Overall model significance was evaluated using the likelihood ratio test, and model explanatory power was summarized using Nagelkerke *R*^2^.

BMI was not included in the GNRI models because the ratio of current body weight to ideal body weight is a structural component of the GNRI formula [18]. Adjustment for BMI would therefore introduce circularity between a covariate and the outcome. For PNI and CONUT, BMI was also excluded from the primary models because contemporaneously measured BMI could be an intermediate factor between masticatory performance and the laboratory components of these indices. Because temporal ordering cannot be established in a cross-sectional study, exploratory BMI-adjusted models are presented in Appendix Table A2 and Table A3 and should not be interpreted as estimates of a BMI-independent effect.

The primary GNRI Model 1 included 39 nutritional-risk events and four parameters, corresponding to an events-per-variable (EPV) [38] of 9.75. Firth’s penalized-likelihood logistic regression with profile penalized-likelihood CIs was therefore performed as a sensitivity analysis for this model using the logistf package in R. Because Model 2 contained more parameters and had a lower EPV, its estimates were regarded as exploratory and interpreted cautiously. Similarity between conventional and Firth estimates was considered supportive, but not conclusive, evidence of estimate stability.

Pairwise agreement among the GNRI-, PNI-, and CONUT-based classifications was assessed using Cohen’s κ coefficients, with 95% CIs calculated using the large-sample (asymptotic) standard error method [39]. Differences in the magnitude of associations across the three nutritional indices were not formally tested. Consequently, differences in statistical significance between the index-specific models were not interpreted as evidence that the associations differed in magnitude.

Because the PNI and CONUT analyses, Model 2 analyses, and BMI-adjusted analyses were secondary or exploratory, their *p*-values were interpreted as nominal. All statistical tests were two-sided, and *p* < 0.05 was considered statistically significant.

## 3. Results

### 3.1. Study Participants

Of the 187 patients recruited, one was excluded because the current-to-ideal body weight ratio component of the GNRI could not be validly assessed owing to lower-limb amputation, and seven were excluded because of missing data for one or more variables required for the multivariable analyses. The remaining 179 participants had complete data and were included in the final analysis.

### 3.2. Participant Characteristics

Participant characteristics are summarized in Table 1. The mean age was 74.1 ± 6.9 years, and 109 participants (60.9%) were female. The median masticatory performance was 154.0 mg/dL (IQR, 122.5–190.0). Nutritional risk was identified in 39 participants (21.8%) using the GNRI, 87 (48.6%) using the PNI, and 56 (31.3%) using the CONUT score. No participant had a CONUT score of 5 or higher.

### 3.3. Participant Characteristics According to GNRI-Defined Nutritional Risk

Participant characteristics according to GNRI-defined nutritional risk are presented in Table 2. Participants classified as being at nutritional risk had lower masticatory performance than those without nutritional risk (median [IQR], 126.0 [87.5–172.0] vs. 162.0 [131.0–196.3] mg/dL; *p* = 0.001), as well as lower BMI (mean ± SD, 19.4 ± 3.2 vs. 23.4 ± 3.0 kg/m^2^; *p* < 0.001) and lower serum albumin concentration (median [IQR], 3.9 [3.8–4.1] vs. 4.2 [4.1–4.4] g/dL; *p* < 0.001). No statistically significant between-group differences were observed for the remaining demographic, clinical, oral, dietary, or socioeconomic variables.

### 3.4. Associations Between Masticatory Performance and Nutritional Risk

The results of the multivariable logistic regression analyses are presented in Table 3. No substantial multicollinearity was identified among the explanatory variables in either model for any of the three outcomes; all VIFs were <2.

For GNRI-defined nutritional risk (Table 3a), higher masticatory performance was associated with lower odds of being classified as at nutritional risk in the primary parsimonious model (Model 1: OR per 10 mg/dL increase, 0.89; 95% CI, 0.82–0.95; *p* = 0.001). A similar estimate was obtained in the exploratory additionally adjusted model (Model 2: OR, 0.89; 95% CI, 0.82–0.96; *p* = 0.002). The likelihood ratio tests yielded *p* = 0.005 for Model 1 and *p* = 0.023 for Model 2. No other covariate was statistically significantly associated with GNRI-defined nutritional risk in either model.

In the Firth’s penalized-likelihood sensitivity analysis using the Model 1 variable set (Appendix Table A1), the estimate for masticatory performance was similar to that obtained using conventional logistic regression (OR per 10 mg/dL increase, 0.89; 95% CI, 0.83–0.95; *p* = 0.001). None of the other covariates was statistically significant.

For PNI-defined nutritional risk (Table 3b), masticatory performance was not statistically significantly associated with nutritional risk in Model 1 (OR per 10 mg/dL increase, 0.95; 95% CI, 0.90–1.01; *p* = 0.091). In the exploratory Model 2, the coefficient for masticatory performance yielded a nominal *p*-value of 0.047 (OR, 0.94; 95% CI, 0.89–1.00). Age also yielded a nominal *p*-value of 0.047 in Model 2 (OR per 1-year increase, 1.05; 95% CI, 1.00–1.10). The likelihood ratio tests were not statistically significant for either PNI model (Model 1, *p* = 0.258; Model 2, *p* = 0.337).

For CONUT-defined nutritional risk (Table 3c), masticatory performance was not statistically significantly associated with nutritional risk in either model (Model 1: OR per 10 mg/dL increase, 1.00; 95% CI, 0.95–1.06; *p* = 0.941; Model 2: OR, 0.99; 95% CI, 0.93–1.05; *p* = 0.656). In the exploratory Model 2, female sex showed a nominal inverse association with CONUT-defined nutritional risk (OR, 0.46; 95% CI, 0.22–0.97; *p* = 0.041). Higher estimated energy intake also showed a borderline inverse association (OR per 100 kcal/day increase, 0.94; 95% CI, 0.88–1.00; *p* = 0.050; unrounded *p* = 0.0497). The likelihood ratio tests were not statistically significant for either CONUT model (Model 1, *p* = 0.292; Model 2, *p* = 0.099).

In the exploratory BMI-adjusted analyses, masticatory performance was not statistically significantly associated with PNI-defined nutritional risk in either model (Model 1: OR, 0.96; 95% CI, 0.90–1.01; *p* = 0.131; Model 2: OR, 0.95; 95% CI, 0.89–1.01; *p* = 0.088). Corresponding estimates for CONUT-defined nutritional risk were also not statistically significant (Model 1: OR, 1.01; 95% CI, 0.96–1.07; *p* = 0.678; Model 2: OR, 1.00; 95% CI, 0.94–1.07; *p* = 0.961). Full results are presented in Appendix B (Table A2 and Table A3).

No formal statistical comparison of the masticatory-performance coefficients across the GNRI-, PNI-, and CONUT-based models was performed.

The linearity of the association between each continuous explanatory variable and the logit of each outcome was assessed using the Box–Tidwell approach; the results are presented in Appendix C (Table A4 and Table A5).

### 3.5. Pairwise Agreement Among Nutritional Risk Classifications

Pairwise agreement among the three nutritional risk classifications is presented in Table 4. Agreement was fair between the GNRI and PNI classifications (κ = 0.342; 95% CI, 0.202–0.481) and between the GNRI and CONUT classifications (κ = 0.278; 95% CI, 0.110–0.445). Agreement between the PNI and CONUT classifications was moderate (κ = 0.424; 95% CI, 0.291–0.558).

## 4. Discussion

This cross-sectional study examined the association between objectively assessed masticatory performance and nutritional risk in older adults wearing removable dentures using the GNRI, PNI, and CONUT score. Lower masticatory performance was consistently associated with higher GNRI-defined nutritional risk in the primary and sensitivity analyses, whereas comparable associations were not consistently observed for PNI- or CONUT-defined risk. Because the magnitudes of the associations were not formally compared across indices, these findings should not be interpreted as evidence that masticatory performance was more strongly associated with GNRI than with PNI or CONUT.

For GNRI-defined nutritional risk, the estimate for masticatory performance was essentially unchanged between the primary parsimonious model (Model 1) and the exploratory additionally adjusted model (Model 2). However, because some variables in Model 2 may lie on the pathway between masticatory performance and nutritional risk rather than act solely as confounders, Model 2 should be interpreted as exploratory. Firth’s penalized-likelihood logistic regression yielded a similar estimate to conventional logistic regression (Appendix Table A1), supporting the stability of the primary finding, although residual confounding and causality cannot be addressed in this cross-sectional design. The Box–Tidwell assessment did not indicate a meaningful departure from linearity (Appendix Table A4) for masticatory performance in the primary model.

One possible explanation for the GNRI finding lies in the composition of the index. GNRI combines serum albumin concentration with the ratio of current to ideal body weight, as specified in the original GNRI formula [18], and therefore reflects both serum albumin and low body weight relative to ideal body weight, rather than directly measured weight loss. These components may capture cumulative nutrition-related changes associated with impaired oral function and altered food selection. The finding is broadly consistent with a recent study reporting associations among masticatory function, food variety, and GNRI [41]. However, this interpretation remains speculative because the magnitudes of the associations across GNRI, PNI, and CONUT were not formally compared. A formal comparison would require a statistical model that incorporates the three nutritional indices simultaneously and tests an interaction between nutritional index and masticatory performance. Such an analysis would also require adequate sample size to detect an interaction effect, which generally requires greater statistical power than testing a main effect. Therefore, the present findings should be interpreted as hypothesis-generating rather than as evidence of a formally established difference in association strength among the three indices.

In the primary parsimonious models, masticatory performance was not significantly associated with PNI- or CONUT-defined nutritional risk. The coefficient for masticatory performance reached nominal significance only in the exploratory PNI Model 2, for which the overall likelihood ratio test was not significant, and should not be regarded as consistent evidence of an association. Differences in index composition may partly explain the observed pattern. PNI combines serum albumin with lymphocyte count, whereas CONUT combines serum albumin, lymphocyte count, and total cholesterol. Lymphocyte count and cholesterol are influenced by immunological, inflammatory, and metabolic factors beyond dietary intake [42,43]. Such variability may have reduced the ability of PNI and CONUT to capture oral-function-related nutritional variation in this relatively healthy outpatient cohort. Consistent with these compositional differences, agreement among the three classifications was only fair to moderate, indicating that they do not identify nutritional risk interchangeably in this population.

In exploratory analyses prompted by reported variation in PNI and CONUT across BMI categories [44], additional adjustment for BMI did not materially change the estimates for masticatory performance (Appendix Table A2 and Table A3). Because BMI was measured contemporaneously and may be an intermediate factor rather than solely a confounder, these analyses should be interpreted cautiously. A few other coefficients reached nominal significance, including age in the PNI model and female sex and estimated energy intake in the CONUT model. Given the secondary nature of these analyses, the number of coefficients examined, and the non-significant overall likelihood ratio tests, these findings should be considered hypothesis-generating. The sex-related finding is broadly consistent with reported differences in dietary intake among Japanese older adults [45] but requires confirmation.

A dietary pathway may underlie the association between masticatory performance and GNRI-defined nutritional risk. Reduced masticatory performance can limit consumption of hard or fibrous foods, such as meat, vegetables, and fruit [2], potentially reducing dietary diversity and nutrient intake [15,16,46]. In the present study, however, estimated total energy intake neither differed between GNRI-defined risk groups nor was associated with GNRI-defined risk in Model 2. This does not exclude a dietary mechanism, because impaired mastication may affect food selection, texture, variety, and nutrient quality in ways not captured by total energy intake alone. Previous studies have similarly linked masticatory performance with frailty independently of measured nutrient intake [47] and suggested that food variety may partly mediate relationships between masticatory function and nutrition-related outcomes [41].

The number of functional teeth, a widely used structural indicator of oral status [48], was not associated with nutritional risk in any model, despite previous reports linking tooth loss to higher nutritional risk, including lower serum albumin levels [15,16]. Tooth count may not adequately represent functional chewing ability, particularly in removable denture wearers. This interpretation is consistent with evidence that improved tooth retention over time was not accompanied by a corresponding improvement in objectively measured masticatory performance [49]. Objective assessment of mastication may therefore provide nutrition-related information beyond conventional structural measures of oral status.

These findings have two main clinical implications. First, within the broader context of oral frailty and its adverse health consequences [50], low masticatory performance identified during routine prosthodontic examination may provide clinically relevant information regarding GNRI-defined nutritional risk and may serve as a signal to consider a more comprehensive nutritional evaluation. The present study did not assess diagnostic accuracy, predictive performance, or the effectiveness of screening and referral pathways, and these issues require prospective investigation. Second, dietary guidance combined with prosthodontic treatment has been reported to improve nutritional biomarkers or dietary intake more effectively than prosthodontic treatment alone [51,52,53]. The present findings therefore support further evaluation of integrated prosthodontic and nutritional care when nutritional improvement is a treatment goal.

The strengths of this study include its focus on a clinically relevant population of older removable denture wearers, objective assessment of masticatory performance, comprehensive evaluation of oral status, and simultaneous assessment of three widely used composite nutritional indices (GNRI, PNI, and CONUT), which allowed the pattern of findings to be examined across multiple nutritional screening tools rather than a single index. Hierarchical regression, complemented by a Firth sensitivity analysis for the primary GNRI model and an assessment of the linearity of continuous predictors using the Box–Tidwell approach, allowed the contribution of potential confounders and key model assumptions to be examined systematically.

Several methodological limitations and potential sources of bias should be acknowledged. The cross-sectional design precludes conclusions regarding temporal direction or causality. Participants were ambulatory patients from a single university dental hospital, which may have introduced selection bias and limits generalizability to the broader community-dwelling population and to care-dependent or institutionalized older adults. Energy intake was self-reported using the BDHQ, and dietary diversity, food texture, and food avoidance were not directly assessed. Masticatory performance was assessed using a single, non-habituated measurement, consistent with prior studies [29,30]. A reliability study of the same test reported lower ICC for a non-habituated measurement (0.708) than for a habituation-preceded measurement (0.924–0.945) [54], suggesting that a single, non-habituated measurement may be associated with greater measurement variability, which should be considered when interpreting the null findings for PNI and CONUT. Perceived economic status was assessed using a non-validated, single-item question. GNRI-defined risk reflects a screening-level classification rather than a comprehensive clinical nutritional evaluation, and may partly reflect its albumin and body-weight components. Complete-case analysis may have introduced bias. The exploratory design, absence of an a priori sample size calculation, and limited number of events reduced the strength of inference, especially for PNI, CONUT, and formal comparisons across indices; this limitation was particularly relevant for Model 2, where the EPV fell well below conventional thresholds. Although a Firth sensitivity analysis supported the stability of the primary Model 1 estimate, this does not resolve the underlying power limitation, and residual confounding cannot be excluded.

Prospective longitudinal studies in larger and more diverse populations are needed to clarify the temporal relationship between masticatory performance and nutritional risk. Future studies should also determine whether objective masticatory assessment adds clinically useful information to established nutritional evaluations and whether integrated prosthodontic and nutritional interventions improve patient outcomes.

## 5. Conclusions

In this cross-sectional study of older adults wearing removable dentures, lower masticatory performance was consistently associated with higher GNRI-defined nutritional risk after adjustment for age, sex, and comorbidity burden, whereas comparable associations were not consistently observed for PNI- or CONUT-defined risk. Because association magnitudes were not formally compared across indices, the findings do not establish that the association was stronger for GNRI than for the other indices. As GNRI incorporates serum albumin and body-weight status, this association should be interpreted as a marker-level relationship with GNRI-defined risk rather than as a diagnosis of malnutrition. Given the cross-sectional design and potential for residual confounding, causality cannot be inferred. Nevertheless, objective assessment of masticatory performance may provide clinically relevant information regarding GNRI-defined nutritional risk in this population and merits further evaluation in larger, more diverse cohorts.

## Figures and Tables

**Table 1:** Characteristics of the study participants.

Characteristic		Overall (*n* = 179)
Demographics		
Age, years		74.1 ± 6.9
Sex, *n* (%)	Male	70 (39.1)
	Female	109 (60.9)
BMI (kg/m^2^)		22.5 ± 3.4
Clinical		
CCI, *n* (%)	0	101 (56.4)
	≥1	78 (43.6)
Estimated energy intake (kcal/day)		1802 [1449, 2277]
Socioeconomic		
Living arrangement, *n* (%)	Living alone	49 (27.4)
	Living with others	130 (72.6)
Perceived economic status, *n* (%)	High	131 (73.2)
	Low	48 (26.8)
Oral		
Masticatory performance (mg/dL)		154.0 [122.5, 190.0]
Denture quality score		74.5 [63.5, 83.3]
Number of functional teeth		18.0 [11.0, 22.0]
Nutritional		
GNRI (continuous)		101.3 [98.3, 105.2]
GNRI categories, *n* (%)	>98 (No nutritional risk)	140 (78.2)
	≤98 (At nutritional risk)	39 (21.8)
PNI (continuous)		50.1 [47.2, 52.3]
PNI categories, *n* (%)	>50 (No nutritional risk)	92 (51.4)
	≤50 (At nutritional risk)	87 (48.6)
CONUT score (ordinal)		1 [0, 2]
CONUT categories, *n* (%)	0–1 (No nutritional risk)	123 (68.7)
	≥2 (At nutritional risk)	56 (31.3)
Albumin (g/dL) ^†^		4.2 [4.0, 4.3]

Data are presented as mean ± standard deviation (SD), median [interquartile range (IQR)], or number (%), as appropriate. CCI, Charlson Comorbidity Index; GNRI, Geriatric Nutritional Risk Index; PNI, Prognostic Nutritional Index; CONUT, Controlling Nutritional Status. Masticatory performance was assessed as the glucose concentration (mg/dL) released from a standardized gummy jelly (gummy jelly test); higher concentrations indicate better masticatory performance. ^†^ Albumin, a shared component of the GNRI, PNI, and CONUT scores, is presented separately for reference.

**Table 2:** Comparison of participant characteristics according to GNRI-defined nutritional risk.

Variable		No Nutritional Risk (GNRI > 98) *n* = 140	At Nutritional Risk (GNRI ≤ 98) *n* = 39	*p*-Value
Age, years		74.1 ± 6.7	74.0 ± 7.9	0.920
BMI (kg/m^2^)		23.4 ± 3.0	19.4 ± 3.2	<0.001
Estimated energy intake (kcal/day)		1803 [1458, 2354]	1762 [1397, 2027]	0.324
Masticatory performance (mg/dL)		162.0 [131.0, 196.3]	126.0 [87.5, 172.0]	0.001
Number of functional teeth		18.0 [12.8, 22.0]	15.0 [8.5, 22.0]	0.439
Denture quality score		76.3 [64.9, 82.6]	70.5 [59.3, 85.3]	0.166
Albumin (g/dL)		4.2 [4.1, 4.4]	3.9 [3.8, 4.1]	<0.001
Sex, *n* (%)	Male	59 (42.1)	11 (28.2)	0.139
	Female	81 (57.9)	28 (71.8)	
CCI, *n* (%)	0	81 (57.9)	20 (51.3)	0.472
	≥1	59 (42.1)	19 (48.7)	
Living arrangement, *n* (%)	Living alone	38 (27.1)	11 (28.2)	1.000
	Living with others	102 (72.9)	28 (71.8)	
Perceived economic status, *n* (%)	High	105 (75.0)	26 (66.7)	0.312
	Low	35 (25.0)	13 (33.3)	

GNRI, Geriatric Nutritional Risk Index; CCI, Charlson Comorbidity Index. Data are presented as mean ± standard deviation (SD), median [interquartile range (IQR)], or number (%), as appropriate. Continuous variables were evaluated for normality using the Shapiro–Wilk test; *p*-values were calculated using Student’s *t*-test for normally distributed data or the Mann–Whitney *U* test for non-normally distributed data, and the chi-square test or Fisher’s exact test for categorical variables, as appropriate.

**Table 3:** Multivariable logistic regression analyses for nutritional risk defined by (**a**) GNRI, (**b**) PNI, and (**c**) CONUT.

(**a**) Outcome: GNRI-defined nutritional risk				
	**Model 1**		**Model 2**	
	**OR (95% CI)**	***p*** **-Value**	**OR (95% CI)**	***p*** **-Value**
Variables included in both models				
Masticatory performance (per 10 mg/dL increase)	0.89 (0.82–0.95)	0.001	0.89 (0.82–0.96)	0.002
Age (per 1-year increase)	1.00 (0.95–1.06)	0.973	1.01 (0.95–1.06)	0.831
Female sex (vs. male)	1.79 (0.79–4.07)	0.162	1.78 (0.74–4.32)	0.200
CCI ≥ 1 (vs. 0)	1.31 (0.62–2.81)	0.480	1.29 (0.60–2.81)	0.514
Additional variables in Model 2 only				
Estimated energy intake (per 100 kcal/day increase)			0.96 (0.90–1.03)	0.281
Living with others (vs. living alone)			1.27 (0.54–3.04)	0.585
Low perceived economic status (vs. high)			1.57 (0.68–3.61)	0.289
Number of functional teeth (per 1-tooth increase)			1.02 (0.96–1.08)	0.597
Denture quality score (per 1-point increase)			0.98 (0.95–1.01)	0.163
Likelihood ratio test, *p*-value	0.005		0.023	
Nagelkerke *R*^2^	0.124		0.157	
(**b**) Outcome: PNI-defined nutritional risk				
	**Model 1**		**Model 2**	
	**OR (95% CI)**	***p*** **-Value**	**OR (95% CI)**	***p*** **-Value**
Variables included in both models				
Masticatory performance (per 10 mg/dL increase)	0.95 (0.90–1.01)	0.091	0.94 (0.89–1.00)	0.047
Age (per 1-year increase)	1.04 (0.99–1.08)	0.114	1.05 (1.00–1.10)	0.047
Female sex (vs. male)	0.96 (0.51–1.79)	0.897	0.97 (0.50–1.91)	0.939
CCI ≥ 1 (vs. 0)	0.82 (0.44–1.51)	0.519	0.83 (0.44–1.56)	0.571
Additional variables in Model 2 only				
Estimated energy intake (per 100 kcal/day increase)			0.98 (0.93–1.03)	0.341
Living with others (vs. living alone)			1.40 (0.69–2.87)	0.350
Low perceived economic status (vs. high)			1.17 (0.59–2.35)	0.652
Number of functional teeth (per 1-tooth increase)			1.05 (1.00–1.10)	0.076
Denture quality score (per 1-point increase)			0.99 (0.96–1.01)	0.274
Likelihood ratio test, *p*-value	0.258		0.337	
Nagelkerke *R*^2^	0.039		0.074	
(**c**) Outcome: CONUT-defined nutritional risk				
	**Model 1**		**Model 2**	
	**OR (95% CI)**	***p*** **-Value**	**OR (95% CI)**	***p*** **-Value**
Variables included in both models				
Masticatory performance (per 10 mg/dL increase)	1.00 (0.95–1.06)	0.941	0.99 (0.93–1.05)	0.656
Age (per 1-year increase)	1.02 (0.97–1.07)	0.464	1.04 (0.99–1.09)	0.162
Female sex (vs. male)	0.63 (0.32–1.21)	0.165	0.46 (0.22–0.97)	0.041
CCI ≥ 1 (vs. 0)	1.47 (0.77–2.84)	0.246	1.53 (0.78–3.01)	0.220
Additional variables in Model 2 only				
Estimated energy intake (per 100 kcal/day increase)			0.94 (0.88–1.00)	0.050
Living with others (vs. living alone)			0.67 (0.31–1.43)	0.298
Low perceived economic status (vs. high)			0.83 (0.39–1.80)	0.642
Number of functional teeth (per 1-tooth increase)			1.06 (1.00–1.12)	0.060
Denture quality score (per 1-point increase)			0.99 (0.96–1.01)	0.322
Likelihood ratio test, *p*-value	0.292		0.099	
Nagelkerke *R*^2^	0.038		0.111	

GNRI, Geriatric Nutritional Risk Index; PNI, Prognostic Nutritional Index; CONUT, Controlling Nutritional Status; OR, odds ratio; CI, confidence interval; CCI, Charlson Comorbidity Index. Model 1 is the primary parsimonious model; Model 2 is the exploratory additionally adjusted model. Nutritional risk was coded as 1, and no nutritional risk was coded as 0. Variables listed under “Additional variables in Model 2 only” were not included in Model 1. The *p*-value for estimated energy intake in Model 2 for CONUT-defined nutritional risk is displayed as 0.050 after rounding (unrounded *p* = 0.0497). BMI was not included in the primary model because it may represent a mediator, rather than a confounder, of the association between masticatory performance and nutritional status in this cross-sectional design (see Appendix Table A2 and Table A3 for BMI-adjusted exploratory models).

**Table 4:** Pairwise agreement between nutritional risk classifications: (**a**) GNRI and PNI; (**b**) GNRI and CONUT; (**c**) PNI and CONUT.

(**a**). GNRI- and PNI-defined nutritional risk			
	**PNI > 50**	**PNI ≤ 50**	**Total**
GNRI > 98	87	53	140
GNRI ≤ 98	5	34	39
Total	92	87	179
κ = 0.342 (95% CI, 0.202–0.481)			
(**b**). GNRI- and CONUT-defined nutritional risk			
	**CONUT 0–1**	**CONUT ≥ 2**	**Total**
GNRI > 98	106	34	140
GNRI ≤ 98	17	22	39
Total	123	56	179
κ = 0.278 (95% CI, 0.110–0.445)			
(**c**). PNI- and CONUT-defined nutritional risk			
	**CONUT 0–1**	**CONUT ≥ 2**	**Total**
PNI > 50	82	10	92
PNI ≤ 50	41	46	87
Total	123	56	179
κ = 0.424 (95% CI, 0.291–0.558)			

GNRI, Geriatric Nutritional Risk Index; PNI, Prognostic Nutritional Index; CONUT, Controlling Nutritional Status; CI, confidence interval. Agreement was assessed using Cohen’s kappa coefficient (κ) and interpreted according to the classification: 0.21–0.40, fair; 0.41–0.60, moderate [40].

## Data Availability

The data presented in this study are available on request from the corresponding author due to privacy and ethical restrictions.

## Abbreviations

The following abbreviations are used in this manuscript:

BDHQ	Brief-type self-administered diet history questionnaire
BMI	Body mass index
CCI	Charlson Comorbidity Index
CI	Confidence interval
CONUT	Controlling Nutritional Status
EPV	Events per variable
GLIM	Global Leadership Initiative on Malnutrition
GNRI	Geriatric Nutritional Risk Index
ICC	Intraclass correlation coefficient
IQR	Interquartile range
MNA	Mini Nutritional Assessment
OR	Odds ratio
PNI	Prognostic Nutritional Index
SD	Standard deviation
STROBE	Strengthening the Reporting of Observational Studies in Epidemiology
VIF	Variance inflation factor

## Appendix A

Firth’s penalized-likelihood logistic regression was applied to the primary parsimonious GNRI model (Model 1: masticatory performance, age, sex, and CCI) as a sensitivity analysis, given the limited number of GNRI-defined nutritional-risk events relative to the number of parameters (EPV = 9.75). Because Firth’s method reduces small-sample bias rather than resolving inadequate statistical power, similarity between the Firth and conventional estimates was interpreted as supportive of, but not conclusive evidence for, estimate stability.

**Table A1:** Sensitivity analysis using Firth’s penalized likelihood logistic regression for GNRI-defined nutritional risk (parsimonious Model 1).

Variable	OR (95%CI)	*p*-Value
Masticatory performance (per 10 mg/dL increase)	0.89 (0.83–0.95)	0.001
Age (per 1-year increase)	1.00 (0.95–1.05)	0.972
Female sex (vs. male)	1.73 (0.80–3.97)	0.168
CCI ≥ 1 (vs. 0)	1.30 (0.62–2.75)	0.487

OR, odds ratio; CI, confidence interval (profile penalized-likelihood CI). Nutritional risk was coded as 1, and no nutritional risk was coded as 0.

## Appendix B

### Appendix B.1. Exploratory BMI-Adjusted Analyses for PNI-Defined Nutritional Risk

As an exploratory analysis, BMI was additionally included in Model 1 and Model 2 for PNI-defined nutritional risk, to examine how the estimated associations changed after conditioning on BMI. Because BMI may lie on the pathway between masticatory performance and nutritional risk, it was excluded from the primary models, and these results should be interpreted as exploratory rather than as estimates of the primary association.

**Table A2:** Exploratory BMI-adjusted logistic regression analyses for PNI-defined nutritional risk.

	Model 1 + BMI		Model 2 + BMI	
	OR (95% CI)	*p*-Value	OR (95% CI)	*p*-Value
Variables included in both models				
Masticatory performance (per 10 mg/dL increase)	0.96 (0.90–1.01)	0.131	0.95 (0.89–1.01)	0.088
Age (per 1-year increase)	1.04 (0.99–1.09)	0.127	1.05 (1.00–1.10)	0.064
Female sex (vs. male)	0.81 (0.42–1.57)	0.541	0.84 (0.41–1.71)	0.622
BMI (per 1 kg/m^2^ increase)	0.84 (0.76–0.93)	0.001	0.84 (0.76–0.93)	0.001
CCI ≥ 1 (vs. 0)	0.82 (0.43–1.57)	0.554	0.83 (0.43–1.60)	0.577
Additional variables in Model 2 + BMI				
Estimated energy intake (per 100 kcal/day increase)			0.98 (0.92–1.03)	0.356
Living with others (vs. living alone)			1.38 (0.65–2.93)	0.396
Low perceived economic status (vs. high)			1.19 (0.58–2.45)	0.629
Number of functional teeth (per 1-tooth increase)			1.04 (0.99–1.10)	0.102
Denture quality score (per 1-point increase)			0.98 (0.95–1.01)	0.131
Likelihood ratio test, *p*-value	0.003		0.011	
Nagelkerke *R*^2^	0.128		0.161	

PNI, Prognostic Nutritional Index; OR, odds ratio; CI, confidence interval. Model 1 is the primary parsimonious model; Model 2 is the exploratory additionally adjusted model. Nutritional risk was coded as 1, and no nutritional risk was coded as 0. Variables listed under “Additional variables in Model 2 + BMI” were not included in Model 1 + BMI.

### Appendix B.2. Exploratory BMI-Adjusted Analyses for CONUT-Defined Nutritional Risk

The same exploratory BMI-adjusted analysis was performed for CONUT-defined nutritional risk, for the same rationale as in Appendix B.1.

**Table A3:** Exploratory BMI-adjusted logistic regression analyses for CONUT-defined nutritional risk.

	Model 1 + BMI		Model 2 + BMI	
	OR (95% CI)	*p*-Value	OR (95% CI)	*p*-Value
Variables included in both models				
Masticatory performance (per 10 mg/dL increase)	1.01 (0.96–1.07)	0.678	1.00 (0.94–1.07)	0.961
Age (per 1-year increase)	1.02 (0.97–1.07)	0.519	1.03 (0.98–1.09)	0.261
Female sex (vs. male)	0.50 (0.25–1.01)	0.053	0.35 (0.16–0.79)	0.011
BMI (per 1 kg/m^2^ increase)	0.81 (0.72–0.91)	<0.001	0.80 (0.71–0.90)	<0.001
CCI ≥ 1 (vs. 0)	1.56 (0.79–3.10)	0.201	1.60 (0.79–3.28)	0.194
Additional variables in Model 2 + BMI				
Estimated energy intake (per 100 kcal/day increase)			0.94 (0.88–1.00)	0.050
Living with others (vs. living alone)			0.61 (0.27–1.37)	0.231
Low perceived economic status (vs. high)			0.81 (0.36–1.82)	0.615
Number of functional teeth (per 1-tooth increase)			1.06 (1.00–1.12)	0.072
Denture quality score (per 1-point increase)			0.98 (0.95–1.01)	0.139
Likelihood ratio test, *p*-value	0.001		0.001	
Nagelkerke *R*^2^	0.149		0.220	

CONUT, Controlling Nutritional Status; OR, odds ratio; CI, confidence interval. Model 1 is the primary parsimonious model; Model 2 is the exploratory additionally adjusted model. Nutritional risk was coded as 1, and no nutritional risk was coded as 0. Variables listed under “Additional variables in Model 2 + BMI” were not included in Model 1 + BMI. The estimated energy intake in Model 2 + BMI for CONUT-defined nutritional risk did not reach statistical significance at the two-sided α = 0.05 level (unrounded *p* = 0.0504).

## Appendix C

The linearity of the logit was assessed for all continuous explanatory variables using the Box–Tidwell approach (Appendix Table A4). No significant departure from linearity was observed for masticatory performance, age, estimated energy intake, or denture quality score in any model. In the exploratory BMI-adjusted CONUT Model 1 (Appendix Table A3), the BMI × log(BMI) term was significant (*p* = 0.042), but the likelihood ratio test for the nonlinear term was not (*p* = 0.067); BMI was therefore retained as linear.

Because seven participants had zero functional teeth, this variable was instead evaluated using a four-category sensitivity analysis (0, 1–9, 10–19, ≥20 teeth). The resulting ORs for masticatory performance were essentially unchanged from the original models (Appendix Table A5), indicating that the linear treatment of functional teeth did not materially affect the primary association.

**Table A4:** Box–Tidwell interaction term *p*-values by model.

Model	Masticatory Performance	Age	Energy Intake	Denture Quality	BMI
GNRI Model 1	0.600	0.292	—	—	—
GNRI Model 2	0.137	0.120	0.056	0.144	—
PNI Model 1	0.246	0.157	—	—	—
PNI Model 2	0.307	0.155	0.455	0.452	—
CONUT Model 1	0.671	0.409	—	—	—
CONUT Model 2	0.667	0.348	0.924	0.624	—
PNI Model 1 (BMI-adjusted, Appendix B.1)	0.349	0.103	—	—	0.689
PNI Model 2 (BMI-adjusted, Appendix B.1)	0.383	0.108	0.233	0.150	0.470
CONUT Model 1 (BMI-adjusted, Appendix B.2)	0.392	0.186	—	—	0.042
CONUT Model 2 (BMI-adjusted, Appendix B.2)	0.316	0.141	0.464	0.507	0.098

GNRI, Geriatric Nutritional Risk Index; PNI, Prognostic Nutritional Index; CONUT, Controlling Nutritional Status; CI, confidence interval.

**Table A5:** Odds ratios for masticatory performance: original models vs. models with categorized functional teeth.

Model	Original OR (95% CI), *p*-Value	Categorized Functional Teeth OR (95% CI), *p*-Value
GNRI Model 2	0.89 (0.82–0.96), 0.002	0.88 (0.82–0.95), 0.002
PNI Model 2	0.94 (0.89–1.00), 0.047	0.94 (0.89–1.00), 0.055
CONUT Model 2	0.99 (0.93–1.05), 0.656	0.99 (0.93–1.05), 0.691
PNI Model 2 (BMI-adjusted)	0.95 (0.89–1.01), 0.088	0.95 (0.89–1.01), 0.110
CONUT Model 2 (BMI-adjusted)	1.00 (0.94–1.07), 0.961	1.01 (0.94–1.07), 0.876

GNRI, Geriatric Nutritional Risk Index; PNI, Prognostic Nutritional Index; CONUT, Controlling Nutritional Status; OR, odds ratio; CI, confidence interval.
